# Early induction of hepatic deiodinase type 1 inhibits hepatosteatosis during NAFLD progression

**DOI:** 10.1016/j.molmet.2021.101266

**Published:** 2021-06-05

**Authors:** Eveline Bruinstroop, Jin Zhou, Madhulika Tripathi, Winifred W. Yau, Anita Boelen, Brijesh Kumar Singh, Paul M. Yen

**Affiliations:** 1 Laboratory of Hormonal Regulation, Cardiovascular and Metabolic Disorders, Duke-NUS Medical School, 8 College Road, Singapore 169857, Singapore; 2 Department of Endocrinology & Metabolism, Amsterdam Gastroenterology, Endocrinology & Metabolism, Amsterdam UMC, University of Amsterdam, Meibergdreef 9, 1105 AZ Amsterdam, the Netherlands; 3 Duke Molecular Physiology Institute, Duke University School of Medicine, 300 N Duke St, Durham, NC 27701, USA; 4 Endocrinology, Diabetes, and Metabolism Division, Duke University School of Medicine, 300 N Duke St, Durham, NC 27701, USA; 5 Endocrine Laboratory, Department of Clinical Chemistry, Amsterdam Gastroenterology, Endocrinology & Metabolism, Amsterdam UMC, University of Amsterdam, Meibergdreef 9, 1105 AZ Amsterdam, The Netherlands

**Keywords:** Liver, Steatosis, Thyroid, NAFLD, Deiodinase, NASH

## Abstract

**Objective:**

Nonalcoholic fatty liver disease (NAFLD) comprises a spectrum ranging from hepatosteatosis to progressive nonalcoholic steatohepatitis that can lead to cirrhosis. Humans with low levels of prohormone thyroxine (T_4_) have a higher incidence of NAFLD, and thyroid hormone treatment is very promising in all patients with NAFLD. Deiodinase type 1 (Dio1) is a hepatic enzyme that converts T_4_ to the bioactive T_3_ and therefore regulates thyroid hormone availability within hepatocytes. We investigated the role of this intrahepatic regulation during the progression of NAFLD.

**Methods:**

We investigated hepatic thyroid hormone metabolism in two NAFLD models: wild-type mice fed a Western diet with fructose and *Lepr*^db^ mice fed a methionine- and choline-deficient diet. AAV8-mediated liver-specific Dio1 knockdown was employed to investigate the role of Dio1 during the progression of NAFLD. Intrahepatic thyroid hormone levels, deiodinase activity, and metabolic parameters were measured.

**Results:**

Dio1 expression and activity were increased in the early stages of NAFLD and were associated with an increased T_3_/T_4_ ratio. Prevention of this increase by AAV8-mediated liver-specific Dio1 knockdown increased hepatic triglycerides and cholesterol and decreased the pACC/ACC ratio and acylcarnitine levels, suggesting there was lower β-oxidation. Dio1 siRNA KD in hepatic cells treated with fatty acids showed increased lipid accumulation and decreased oxidative phosphorylation.

**Conclusion:**

Hepatic Dio1 gene expression was modulated by dietary conditions, was increased during hepatosteatosis and early NASH, and regulated hepatic triglyceride content. These early adaptations likely represent compensatory mechanisms that reduce hepatosteatosis and prevent NASH progression.

## Highlights

• Low thyroid hormone action is implicated in the pathogenesis of NAFLD.
• Intracellular thyroid hormone concentrations are regulated by deiodinases.
• Increased deiodinase 1 was found in early NAFLD increasing T_3_/T_4_ ratio.
• AAV8 mediated liver specific knockdown of deiodinase 1 increases steatosis.

## Graphical abstract

**Figure:**
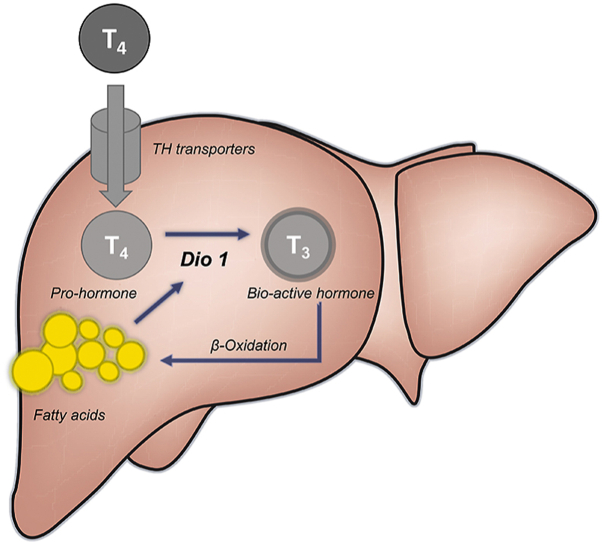

## 1 Introduction

Nonalcoholic fatty liver disease (NAFLD) comprises a spectrum of diseases ranging from simple steatosis in the liver to steatohepatitis (NASH) with inflammation and fibrosis. NAFLD affects approximately 25% of the adult population worldwide, and its rise has been associated with the recent pandemic of obesity and diabetes [1]. Currently, there are no approved drugs for the treatment of NAFLD. Thus, there is an urgent need for the development of new therapies. Recently, thyroid hormone (TH) and TH-analogs (thyromimetics) have shown to be effective therapies for hepatosteatosis and NASH [[2], [3], [4], [5], [6]]. However, the physiological basis of their effects on NAFLD is not well understood at present.

TH stimulates lipophagy, β-oxidation of fatty acids, and oxidative phosphorylation in the liver [4,7]. Previous studies demonstrated that both hypothyroidism and thyroid hormone receptor β mutations in mice and humans increase NAFLD risk [8,9]. Further, lower serum levels of prohormone thyroxine (T_4_), including those within the normal range, increase NAFLD prevalence [10]. However, serum levels of T_4_ are not the only factor determining intrahepatic concentrations of the bioactive triiodothyronine (T_3_), which binds to the nuclear hormone receptor β (TRβ) and causes transcriptional activation of T_3_ target genes. Intrahepatic concentrations of the prohormone T_4_ and the bioactive form of TH (i.e., T_3_) are tightly regulated by deiodinases. There are three deiodinases: deiodinase type1 (Dio1), deiodinase type 2 (Dio2) and deiodinase type 3 (Dio3). These are all selenoenzymes of which Dio1 and Dio3 are expressed in hepatocytes. Dio1 is responsible for outer and inner ring deiodination of the thyroid hormone and is therefore involved in T_3_ production and rT_3_ clearance. Dio3 regulates T_3_ and T_4_ conversion to the inert metabolites T_2_ and rT_3,_ respectively. The expression of hepatic Dio1 is influenced by cytokines and nutritional status, and it is markedly up-regulated by T_3_ [2,11]. Previous research has found low levels of Dio1 expression in the livers of mice after acute and chronic inflammation, as well as rodents and patients with NASH [2,12,13]. In this study, we examined the role of intrahepatic regulation of the thyroid hormone by Dio1 during the different phases of NAFLD progression.

## 2 Materials and methods

### 2.1 General

All mice were maintained according to the Guide for the Care and Use of Laboratory Animals [National Institutes of Health (NIH) publication 1.0.0; revised 2011], and experiments were approved by the Singhealth Institutional Animal Care and Use Committee (2015/SHS/1104).

### 2.2 Western diet and fructose model

Ten-week-old male C57Bl/6J mice were fed a Western diet (D12079B; Research Diets), supplemented with 15% weight/volume fructose (Sigma–Aldrich, 57-48-7) in drinking water for 8 or 16 weeks, whereas the control mice received normal chow and tap water for 16 weeks [14].

### 2.3 Lepr^db^ with MCD diet model

Male BKS.Cg-Dock7m+/+LeprdbJ (db/db) mice (Jackson Laboratory 009659) at 12 weeks of age were fed a normal chow diet or an MCD (A02082002BRi, Research Diets) diet for 2, 4, and 8 weeks to produce NASH stages. C57Bl/6J mice (NUSCARE C57BL/6 JInv) fed a normal chow diet served as the control.

### 2.4 Liver-specific Dio1 knockdown

Ten-week-old male C57Bl/6J mice were injected via tail vein with AAV8-ALB-eGFP-mDio1-shRNAmir or AAV8-ALB-eGFP-ctrl-shRNAmir (Lot 181231#13, Vector Biolabs) and fed with NCD for two weeks, followed by a Western diet with fructose in the drinking water or NCD for the following 12 weeks. A small group of mice (n = 3) injected with the control shRNA were fed with NCD for reference purposes only and not used for statistical purposes.

### 2.5 Cell culture

AML12 cells were passaged in DMEM/F12 (cat. 11320-033), 10% FBS, and 1× pen/strep, insulin transferrin selenium. 24 h after plating the cells, a mix of oleic acid 0.6 M and palmitic acid (OAPA) in the above media (with 1% BSA as carrier or only 1% BSA) was added for 24, 48, and 36 h. For siRNA transfection, AML12 cells were trypsinized and mixed with opti-MEM medium (Invitrogen, 31,985,070), containing Lipofectamine RNAimax (Invitrogen, 13,778,150) and Dio1 (ON-TARGET plus Mouse Dio1 (13370) siRNA SMARTPOOL (Dharmacon), or control siRNA (10 nM)) according to the manufacturer's recommendations. 24 h later, OAPA was added for 24 h. The neutral lipid was stained with fluorescent dye BODIPY 493/503 (5 μg/ml) for 10 min. Oxygen consumption was measured at 37 °C using an XF24 extracellular analyzer (Seahorse Bioscience Inc., North Billerica, MA, USA) [15].

### 2.6 Analysis

Triglyceride concentrations in the liver and serum (10010303; Cayman Chemical Company, Ann Arbor, MI) and total cholesterol (ab65390, abcam) were measured with colorimetric kits according to the manufacturer's instructions after chloroform/methanol lipid extraction. Total RNA isolation was performed using an InviTrap Spin Universal RNA kit (Stratec Biomedical), and RT-qPCR was performed as previously described [15], using a QuantiTect SYBR Green PCR kit (Table primers in supplementary methods). Liver TH concentrations (T_4_ and bioactive T_3_) were measured by LC–MS/MS. Deiodinase 1 (Dio1) and 3 (Dio3) activity were measured by the conversion of ^125^I-labeled rT_3_ and T_3,_ respectively, as previously described [12]. For western blot analysis, proteins were separated by SDS–PAGE under reducing conditions and transferred to nitrocellulose membranes. Membranes were blocked with 5% nonfat milk in phosphate-buffered saline with 0.1% Tween 20 (Sigma–Aldrich, P9416; PBST). The blots were incubated overnight at 4 °C with primary antibodies. Immunoblot analysis was performed using an enhanced chemiluminescence procedure (GE Healthcare, RPN2106).

### 2.7 Statistical analysis

For the WDF model, the groups were compared using a one-way ANOVA with a post-hoc Dunnett's multiple comparison test to establish significance between the groups. For the *Lepr*^*db*^ with MCD model, the wild-type mice with an NCD diet were compared with the *Lepr*^*db*^ mice on an NCD diet with an unpaired t-test to establish the effect of the genotype. We investigated the effect of the MCD diet by comparing the *Lepr*^*db*^ on an NCD diet and 2, 4, and 8 weeks of MCD with a one-way ANOVA with a post-hoc Dunnett's multiple comparison test to establish significance between the groups. Dio1LKD-WDF were compared to the control-WDF with an unpaired t-test. Data points lesser than Q1 − 1.5 × IQR or greater than Q3 + 1.5 × IQR were considered outliers and removed from further analysis. Prism 8 was used for the statistical analysis. Data are expressed as mean ± SEM. Significance was established at p < 0.05.

## 3 Results and discussion

### 3.1 Dio1 expression and intrahepatic TH concentrations in mice fed a Western diet and fructose (WDF)

**Figure 1:**
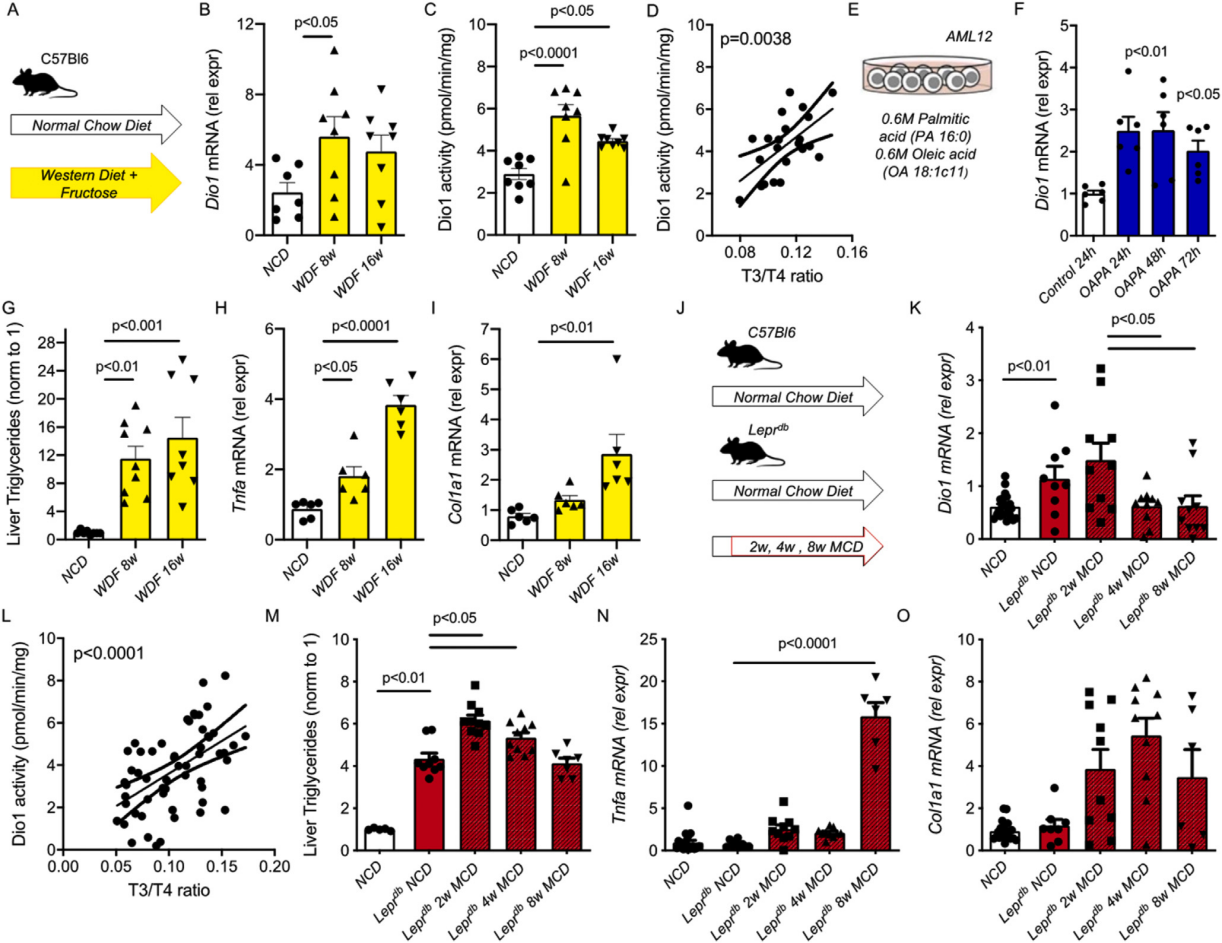
**Dio1 increases early during the progression of NAFLD** (A) Western Diet with 15% fructose in the drinking water (WDF) for 8 and 16 weeks compared to Normal Chow Diet (NCD), (n = 7–8 per group) (B, C) *Dio1mRNA*, Dio1 Activity in the WDF model, (D) Association between Deiodinase 1 activity and liver T_3_/T_4_ ratio in the WDF model (E) Mouse AML12 cell line with oleic acid and palmitic acid (OAPA) (6 wells per group) (F) *Dio1 mRNA* in the AML12 OAPA cell model (G–I) Liver triglycerides, *Tnfa mRNA* and *Col1a1 mRNA* in the WDF model (J) Lepr^db^ model with normal chow diet (NCD) or methionine and choline deficient diet (MCD) compared to C57Bl6 with NCD diet (n = 6–10 per group) (K) *Dio1 mRNA* in the Lepr^db^ model (K) Association between Deiodinase 1 activity and liver T_3_/T_4_ ratio in the Lepr^db^ model (L–O) Liver triglycerides, *Tnfa mRNA* and *Col1a1 mRNA* in the Lepr^db^ model. Data is depicted in mean ± SEM.

To examine intrahepatic TH regulation during the progression of NAFLD, we employed two different NAFLD models to induce hepatosteatosis and NASH. In the first model, we fed mice a Western diet with 15% fructose water (WDF) for 8 and 16 weeks to induce steatosis and early-stage NASH and then compared them with mice fed a normal chow diet (NCD) (Figure 1A) [14]. We observed that hepatic T_4_ decreased in mice fed WDF for 8 (15.3 pmol/g) and 16 (15.1 pmol/g) weeks compared to mice fed NCD (26.0 pmol/g) (Table 1). This decrease in hepatic T_4_ was not explained by a decreased expression of the thyroid hormone transporters *Mct8* and *Mct10*, as we found an increased expression of both transporters in mice fed WDF for 8 weeks, followed by a return to levels similar to those found in mice fed NCD (Table 1) when measured again at 16 weeks. In contrast to the prohormone T_4_, hepatic T_3_ was not significantly different in mice fed WDF for 8 weeks (2.1 pmol/g) and slightly decreased in mice fed WDF for 16 weeks (1.7 pmol/g) compared to mice fed with NCD (2.7 pmol/g) (Table 1). These data suggest that intrahepatic regulation of T_3_ levels during NAFLD progression could be mediated by deiodinases.

**Table 1:** Liver parameters during the progression of NAFLD in the Western Diet with 15% Fructose in the drinking water (left) and the Lepr^db^ model with a methionine and choline deficient diet (MCD) or normal chow diet (NCD) (left). Significance of post-hoc analysis WDF 8w vs. NCD and WDF 16 w vs. NCD (left panel), *Lepr*^*db*^ NCD vs. C57Bl6 NCD, *Lepr*^*db*^ 2w, 4w and 8w post-hoc analysis vs. *Lepr*^*db*^ NCD (right panel). ∗p < 0.05, ∗∗p < 0.01, ∗∗∗p < 0.001, ∗∗∗∗p < 0.0001. Data is depicted in mean ± SEM.

	WDF			Lepr^db^ + MCD				
	NCD	WDF 8w	WDF 16w	Control NCD	Lepr^db^ NCD	Lepr^db^ MCD 2w	Lepr^db^ MCD 4w	Lepr^db^ MCD 8w
Liver T_4_ (pmol/g)	25.96 (2.20)	15.29∗∗∗∗ (1.02)	15.08∗∗∗∗ (0.82)	29.11 (1.03)	17.00∗∗ (1.26)	11.22∗∗ (0.72)	12.22∗∗∗ (0.71)	12.46∗∗∗ (1.31)
Liver T_3_ (pmol/g)	2.66 (0.23)	2.09 (0.32)	1.69∗ (0.06)	2.25 (0.18)	1.99∗∗ (0.09)	1.48∗∗ (0.09)	1.3∗∗∗∗ (0.09)	1.55∗ (0.16)
Liver T_3_/T_4_ ratio	0.10 (0.01)	0.14 (0.02)	0.11 (0.00)	0.08 (0.00)	0.12∗∗∗∗ (0.01)	0.14 (0.00)	0.11 (0.01)	0.11 (0.01)
*Dio3mRNA*	1.90 (0.42)	2.55 (0.71)	2.36 (0.39)	2.09 (0.28)	1.20 (0.29)	2.93∗∗∗ (0.33)	1.64 (0.23)	1.16 (0.27)
Dio3 activity (fmol/min/mg)	0.20 (0.03)	0.14 (0.03)	0.15 (0.01)	0.13 (0.01)	0.05∗∗∗∗ (0.01)	0.07 (0.01)	0.11 (0.03)	0.06 (0.02)
*Thrb mRNA*	0.97 (0.14)	0.94 (0.18)	0.67 (0.10)	3.21 (0.23)	2.46 (0.27)	2.66 (0.30)	1.94 (0.21)	3.86∗ (0.57)
*Mct8 mRNA*	1.07 (0.27)	2.72∗∗ (0.43)	1.94 (0.22)	2.95 (0.32)	2.35 (0.44)	4.89∗ (0.88)	2.94 (0.42)	6.82∗∗ (1.34)
*Mct10 mRNA*	1.31 (0.36)	3.75∗ (1.01)	1.20 (0.26)	2.28 (0.38)	1.16 (0.19)	1.63 (0.34)	1.31 (0.37)	1.57 (0.33)

Next, we examined *Dio1* gene expression, which converts T_4_ to T_3_ in liver cells. *Dio1* mRNA increased more than 2-fold at both 8 and 16 weeks (Figure 1B) in WDF mice. Dio1 activity was measured by the conversion of ^125^I-labeled rT_3_; it increased significantly and was most pronounced at WDF 8 weeks compared to NCD mice (Figure 1C). This increased *Dio1* mRNA and activity was not attributable to increased intrahepatic T_3,_ a known inducer of *Dio1* mRNA expression. Increased Dio1 activity was associated with an increased T_3_/T_4_ ratio for all mice, indicating that it regulated the conversion of the prohormone T_4_ to T_3_ (Figure 1D). We also measured *Dio3* mRNA and Dio3 activity, known to metabolize T_3_ to its inert metabolites, and found they were not significantly different in mice fed WDF *vs.* NCD (Table 1). To further examine *Dio1* mRNA induction during hepatosteatosis, we treated the mouse hepatic cell line, AML12, with a combination of 0.6 M oleic acid and 0.6 M palmitic acid (OAPA) and observed increases in *Dio1* mRNA expression at 24, 48, and 72 h, suggesting that this combination of saturated and monounsaturated fatty acids could induce *Dio1* mRNA expression acutely in a cell autonomous manner (Figure 1E, F). Oleic acid and palmitic acid comprise the most abundant fatty acids in a western diet and, when applied together, cause the greatest lipid accumulation and protect against palmitic acid-induced apoptosis [16]. Nonesterified fatty acids bind to the ligand-binding domain of several nuclear receptors expressed in the liver, including PPAR (α, β, γ), HNF4 (α,γ), retinoid X-receptor (RXR) α, and liver X receptor (LXR) (α,β) [17]. The induction of these transcription factors possibly induces *Dio1* mRNA by a combination of saturated and unsaturated fatty acids.

We measured hepatic triglyceride content and found that it increased more than 10-fold in mice fed WDF for 8 and 16 weeks compared to mice fed NCD (Figure 1G). Hepatic tumor necrosis factor alpha (*Tnfa*) was slightly increased in mice fed WDF for 8 weeks and more than 3-fold in mice fed WDF for 16 weeks (Figure 1H). Alpha-1 type I collagen (*Col1a1*) mRNA significantly increased in mice fed WDF for 16 weeks (Figure 1I). These data suggest that mice fed WDF for 8 weeks had hepatosteatosis and slight inflammation, whereas mice fed WDF for 16 weeks developed early-stage NASH with induction of inflammation and fibrosis marker mRNAs. These changes occurred parallel to increased *Dio1* mRNA expression and activity and were correlated with the T_3_/T_4_ ratio. When taken together, these data suggest that although a previous report determined that Dio1 decreased in late-stage NASH [2], *Dio1* gene expression and activity were increased in hepatosteatosis and early-stage NASH to maintain intrahepatic T3 concentration.

### 3.2 Dio1 expression and intrahepatic TH concentrations in Lepr^db^ mice fed a methionine and choline-deficient diet

To validate this observation in a second NAFLD model, we used *Lepr*^db^ mice that previously developed severe steatosis with only mild inflammation when fed a normal chow diet (NCD) [18]. To induce the NASH phenotype, *Lepr*^db^ were switched after 12 weeks of age to a methionine- and choline-deficient diet (MCD) for 2, 4, and 8 weeks (Figure 1J) [18]. *Lepr*^db^ mice that continued to consume an NCD diet had hepatic T_4_ levels that were 42% lower than wild-type mice on NCD (*Lepr*^db^-NCD T_4_: 17 vs. control-NCD 29.11 pm/g). In this model, increases of the thyroid hormone transporter *Mct8* mRNA were found after 2 and 8 weeks in *Lepr*^db^ mice fed an MCD diet. Next, we investigated intrahepatic T_3_, which decreased by only 12% in *Lepr*^db^-NCD compared to control-NCD (T3: 1.99 vs. 2.25 pmol/g). In this model, we also observed an increase in *Dio1* *mRNA* expression in steatotic livers (Lepr^db^-NCD *vs.* control-NCD) (Figure 1K). *Dio1* *mRNA* decreased below the basal level in *Lepr*^db^ mice fed MCD for 4 and 8 weeks, similar to NASH in rats previously observed by us [2]. The enzyme activity of Dio1 was positively correlated with the T_3_/T_4_ ratio (Figure 1L) again, demonstrating a regulatory role of deiodinases in liver T3 availability.

Triglyceride content in livers from Lepr^db^-NCD mice increased more than 4-fold compared to control-NCD (Figure 1M). Triglyceride content was further increased in *Lepr*^db^ fed MCD for 2 and 4 weeks. *Tnfa* and *Col1a1* mRNA were not significantly different in *Lepr*^db^-NCD and control-NCD mice. However, there was an increased *Tnfa* *mRNA* expression starting at 2 weeks and continuing to 6 weeks, suggesting ongoing inflammation after the initiation of MCD (Figure 1N). *Col1a1* *mRNA* was increased more than two-fold at 2 and 4 weeks and increased 16-fold at 6 weeks (Figure 1). These findings suggest that fibrosis associated with gene expression became more prominent by 8 weeks in the *Lepr*^db^ mice fed MCD. Taken together in this second NAFLD model, an early increase in *Dio1* *mRNA* was also found to be associated with the T_3_/T_4_ ratio.

### 3.3 Liver-specific Dio1 shRNA knockdown in mice fed WDF

**Figure 2:**
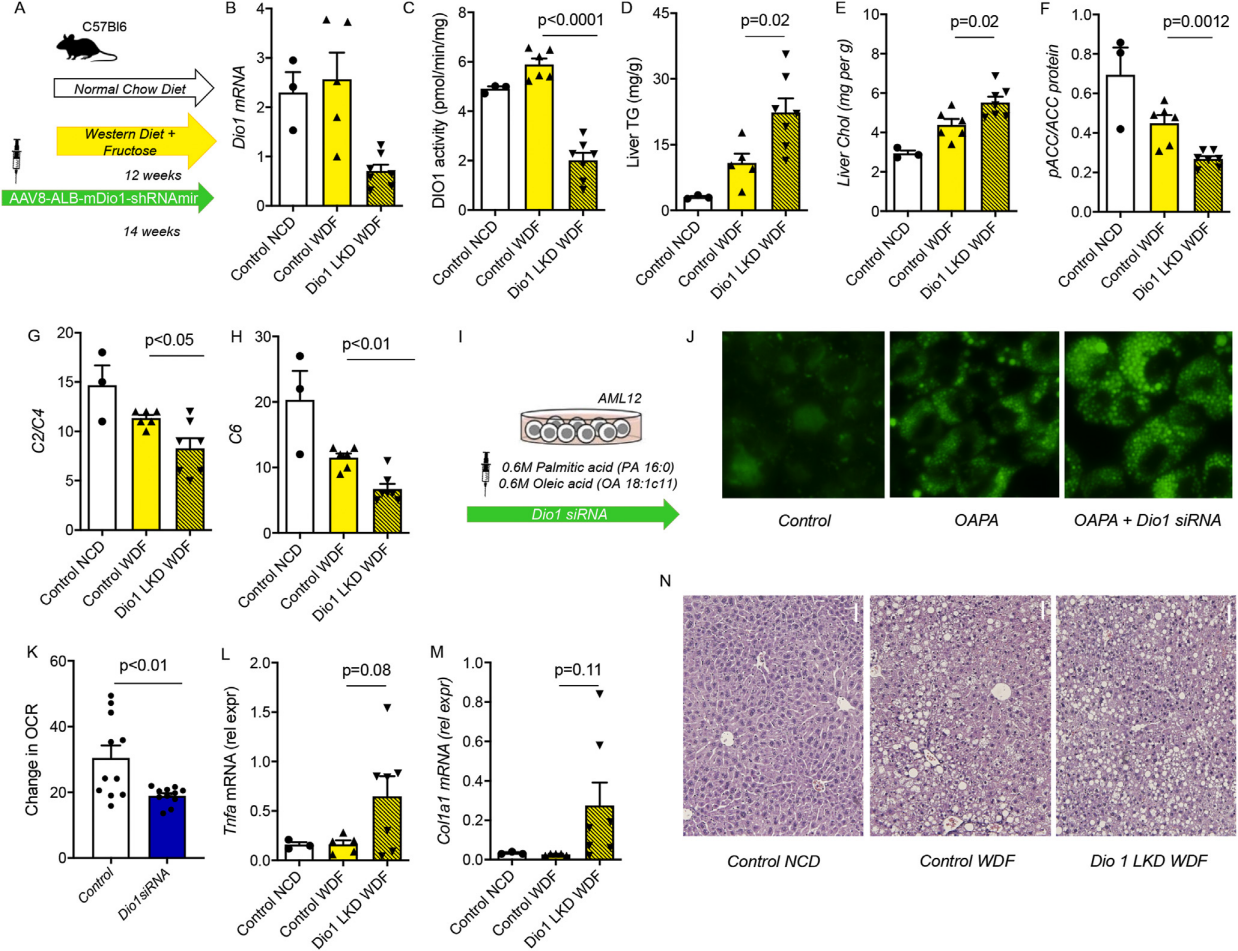
**Dio1 KD increases liver triglycerides and cholesterol.** (A) WDF model with injection of AAV8-Albumin-eGFP-mDio1-shRNAmir (WDF + Dio1 LKD) (n = 7) or AAV8-Albumin-eGFP-ctrl-shRNAmir (WDF + control) (n = 6). For reference, a group of NCD + control shRNA was included (n = 3). (B–G) *Dio1 mRNA* expression (B) DIO1 activity (C), liver triglycerides (TG; D), liver cholesterol (E), densitometric quantification of western blots analyzing pACC/ACC (F), C2/C4 acylcarnitines (G), C6 acylcarnitines (H) in the WDF Dio1 KD model. (I) Schematic representation of *in vitro* experiment utilizing mouse AML12 cell line treated with oleic acid and palmitic acid (OAPA) combined with *Dio1* siRNA knockdown. (J) BODIPY staining of the AML12 cell line combined with OAPA and OAPA with *Dio1 siRNA*. (K) Change in oxygen consumption rate (OCR) after *Dio1* siRNA in AML12 cells. (L–M) *Tnfa mRNA* (L), *Col1a1mRNA* (M) in the WDF Dio1 KD model. (N) Histology of control NCD, control WDF, and Dio1 LKD WDF with TG content on average. Data is depicted in mean ± SEM.

As T_3_ stimulates fatty acid β-oxidation, we investigated whether Dio1 increases during hepatosteatosis and early-stage NASH were protective mechanisms used to maintain an intrahepatic T_3_ concentration to reduce triglyceride accumulation in the liver during nutritional overload. Accordingly, we prevented the early induction of Dio1 by performing a liver-specific knockdown with shRNA against mouse Dio1, cloned under the control of a mouse albumin promoter in an adeno-associated viral vector (AAV8-Albumin-eGFP-mDio1-shRNAmir). Two weeks after tail vein injection of shRNA, mice began consuming WDF or NCD for 12 weeks before sacrifice, which is a transitional period when mice typically convert from hepatosteatosis to early NASH (Figure 2A). The control-WDF group had increased body weight and fat mass compared to control mice fed NCD (i.e., control-NCD). However, there were no significant differences in body weight or fat mass measured by MRI, and food intake was similar for Dio1LKD-WDF and control-WDF mice (data not shown).

We observed that hepatic *Dio1* *mRNA* and Dio1 activity decreased by 72% and 66%, respectively, in Dio1 KD mice fed WDF (Dio1LKD-WDF) compared to control mice fed WDF (control-WDF) 14 weeks after shRNA treatment (Figure 2B–C). Hepatic T_4_ levels were higher in Dio1LKD-WDF (40.6 pmol/g) than control-WDF as would be expected if Dio1 expression/activity were decreased (Table 2). Interestingly, hepatic T_3_ concentrations were not significantly different among the three groups of mice. When we analyzed sera from the mice, we observed increased T_4_ levels in Dio1LKD-WDF (72.4 nmol/l), as compared to the control-WDF group (52.3 nmol/l), which was consistent with findings in the whole-body knockout of Dio1 [19]. Thus, our data showed that a decreased hepatic Dio1 expression and activity from liver-specific KD was sufficient to exert this serum TH profile in mice. Of note, serum T_3_ levels were not significantly different among the three groups of mice. In this experiment, we did not find any differences in *Mct8* and *Mct10* *mRNA* levels among the three groups of mice (Table 2). Taken together, the Dio1LKD-WDF showed a decrease in *Dio1* gene expression and Dio1 activity, resulting in reduced metabolism of intrahepatic T_4_ to T_3._

**Table 2:** Liver and serum parameters in normal chow diet (NCD) with a control shRNA (control-NCD) and western diet with 15% fructose in the drinking water (WDF) with a control shRNA (control-WDF) compared to Dio1 liver-specific knockdown (Dio1LKD-WDF). Significance is shown for WDF control vs. WDF Dio1 LKD. ∗p < 0.05, ∗∗p < 0.01. Data is depicted in mean ± SEM.

	Control-NCD	Control-WDF	Dio1LKD-WDF
T_4_ liver (pmol/g)	41.40 (4.71)	28.12 (2.40)	40.59 (2.81)∗
T_3_ liver (pmol/g)	6.07 (1.01)	6.82 (1.06)	8.86 (2.58)
T_4_ serum (nmol/l)	62.00 (4.04)	52.33 (3.73)	72.43 (3.09)∗∗
T_3_ serum (nmol/l)	1.63 (0.05)	1.68 (0.15)	1.56 (0.10)
*Thrb mRNA*	2.74 (0.55)	1.13 (0.10)	2.15 (0.31)∗
*Mct8 mRNA*	3.67 (1.19)	3.15 (1.41)	4.36 (1.07)
*Mct10 mRNA*	1.23 (0.18)	0.64 (0.09)	1.20 (0.41)

Interestingly, Dio1LKD-WDF showed increased hepatic triglyceride and cholesterol content compared with control-WDF (Figure 2D, E). We thus analyzed hepatic fatty acid metabolism and found Dio1LKD-WDF had a lower pACC/ACC protein ratio than the control-WDF group (Fig. 2F), suggesting increased fatty acid synthesis and decreased β-oxidation of fatty acids. We performed metabolomics of hepatic acylcarnitines as a measure of fatty acid β-oxidation. When comparing control-WDF mice and control-NCD mice, a pattern of decreased short-chain acylcarnitines (C2, C3, C4, C6) and increased medium- and long-chain acylcarnitines (C10:1, C10:2, C12:1, C14:2) emerged, suggesting lower β-oxidation of fatty acids (Supplementary Figure 1). There was a further decrease in the C2/C4 ratio and C6 in Dio1LKD-WDF compared to the control-WDF group (Figure 2G–H). We only observed an increase of the very long acylcarnitine C22:5 (Supplemental Figure 1). We further examined the effects of Dio1 KD by siRNA *in vitro* combined with OAPA treatment in AML12 cells. We measured fat content by BODIPY staining in KD cells and observed increased fat content compared to control cells (Figure 2I–J). Additionally, Dio1 KD cells exhibited decreased oxidative consumption rate by Seahorse analysis, consistent with lower fatty acid β-oxidation (Figure 2K).

Lastly, we noticed a trend toward an increased hepatic expression of *Tnfa* and *Col1a1* *mRNA* in Dio1LKD-WDF compared to control-WDF (Figure 2L, M), with several individual Dio1LKD-WDF showing significant expression of these inflammation and fibrosis markers during this transitional period. In contrast, none of the control-NCD or control-WDF mice had any increases in *Tnfa* and *Col1a1* *mRNA* at 12 weeks. This finding represents differences between experiments in which inflammation is usually induced between 8 and 16 weeks. Histology showed increased fat droplets visible in Dio1LKD-WDF and increased ballooning of hepatocytes (Figure 2N). Serum triglyceride, cholesterol and glucose levels were not significantly altered by Dio1LKD-WDF compared to control-WDF (data not shown).

## 4 Conclusions

Our study demonstrated that hepatic Dio1 expression and activity are increased in early NAFLD, and blocking Dio1 induction by shRNA increased hepatic triglyceride and cholesterol content. These findings may have significant physiological ramifications, as they suggest induction of Dio1 expression and activity during hepatosteatosis, and early NASH may play a preventive role in NAFLD progression. These results help explain our previous finding that levothyroxine (T_4_) can be an effective treatment for hepatosteatosis, as it can convert to T_3_ intrahepatically [2]. It is also possible that patients with a lower Dio1 expression or function may be at a higher risk for developing hepatosteatosis and NASH more rapidly. Decreased Dio1 activity is observed in older age, certain medications (e.g., propranolol and propylthiohuracil), and those with a selenium deficiency. Recently, the first loss-of-function human Dio1 mutation causing changes in thyroid hormone metabolism was described [20]. A Dio1 polymorphism with increased Dio1 activity may protect against hepatosteatosis [21]. Thus, epigenetic and genetic factors could alter Dio1 expression and/or activity, modulating the risk for NAFLD progression. Future studies must determine whether Dio1 overexpression and/or medication-inducing Dio1 activity will have therapeutic potential.

In conclusion, our findings show that hepatic Dio1 expression during early NAFLD is sensitive to nutritional conditions and serves as a metabolic regulator to help reduce hepatosteatosis in early NAFLD.

## Author contributions

**Eveline Bruinstroop:** Conceptualization, Methodology, Formal analysis, Investigation, Writing – Original Draft, Funding acquisition. **Jin Zhou:** Conceptualization, Methodology, Investigation, Writing – Review & Editing. **Madhulika Tripathi:** Investigation, Writing – Review & Editing. **Winifred W. Yau:** Investigation, Writing – Review & Editing. **Anita Boelen:** Conceptualization, Investigation, Writing – Review & Editing. **Brijesh Kumar Singh:** Conceptualization, Methodology, Investigation, Supervision, Writing – Original Draft. **Paul M. Yen:** Conceptualization, Methodology, Supervision, Writing – Original Draft, Funding acquisition.
