# Supplementary material for: Early induction of hepatic deiodinase type 1 inhibits hepatosteatosis during NAFLD progression
Source: Mol Metab. 2021 Jun 5;53:101266. doi: 10.1016/j.molmet.2021.101266 (PMC8237360; doi:10.1016/j.molmet.2021.101266)
Supplement: Multimedia component 1 [file mmc1.pdf]

	Forward	*Reverse*	*Vendor*
Thrb	GAGACTCTAACTTTGAATGGG	CGATCTGAAGACATTAGCAG	Sigma
Mct8 (Slc16a2)	CGTGCACCTGATGAAATATG	GATCATCATGGACATCAAGC	Sigma
Mct10 Slc16a10)	AAGCTCCATCGAGCCTCTGTA	GTCCCAAAATGACCAGTGACG	Sigma
Oatp1c1	CCTTCTCTCTATCTGAGTCACGG	GGGCCATCCTTTACAGTCGG	Sigma
Dio1	ACCCCCGATTCGCCCCTGACAA	ACCAGGGGCCTGCTGCCTTGA	Sigma
Dio3	AAGAAAGTCAAAGGTTGTGG	AAAACGTACAAAAGGGAGTC	Sigma
Col1a1	CGTATCACCAAACTCAGAAG	GAAGCAAAGTTTCCTCCAAG	Sigma
Actin	GTACCACCATGTACCCAGGC	AAGGGTGTAAAACGCAGCTC	Sigma
