# Supplementary material for: Early induction of hepatic deiodinase type 1 inhibits hepatosteatosis during NAFLD progression
Source: Mol Metab. 2021 Jun 5;53:101266. doi: 10.1016/j.molmet.2021.101266 (PMC8237360; doi:10.1016/j.molmet.2021.101266)
Supplement: Multimedia component 2 [file mmc2.pdf]

	NCD Control			WDF Control						WDF Dio1 KD							*WDF control vs WDF Dio1 KD* (p-value)	*WDF control vs NCD control* (p-value)
C2	2730	3687	3107	1210	675	1022	1381	1673	[illegible]	218	264	774	1131	900	852	[illegible]	0,06	0,00
C3	203	140	184	47	102	67	57	80	126	69	78	66	87	234	106	179	0,22	0,00
C4	181	210	277	119	61	82	[illegible]	142	108	38	43	146	121	75	95	106	0,46	0,00
C2/C4	15	18	[illegible]	10	11	12	12	12	11	6	6	5	9	12	9	11	0,03	0,04
C4/C16	6	4	5	2	2	4	5	3	4	4	3	5	3	2	3	3	0,90	0,03
C4-OH	137	266	172	71	40	[illegible]	22	40	[illegible]	37	55	78	84	31	87	27	0,49	0,00
C6	12	27	22	10	12	13	9	13	12	5	6	6	[illegible]	5	6	8	0,00	0,02
C5-OH/C3-DC	25	58	28	23	15	19	15	21	23	21	18	[illegible]	25	19	27	18	0,33	0,04
C8:1-OH/C6:1-DC	29	39	52	146	99	109	121	130	168	163	103	189	234	159	270	152	0,06	0,00
C10:2	2	5	6	9	13	7	6	7	11	9	8	7	9	10	8	6	0,51	0,04
C10:1	5	13	4	32	38	25	21	30	[illegible]	26	22	29	21	22	16	26	0,11	0,00
C12:2	1	4	2	9	6	7	5	7	7	8	5	6	9	7	2	5	0,42	0,00
C12:1	3	2	3	6	5	9	6	7	8	9	3	8	9	7	9	7	0,47	0,00
C12	5	5	6	4	2	2	2	5	4	2	3	3	3	4	4	2	0,64	0,02
C14:2	1	1	1	3	3	2	2	2	3	2	1	1	3	3	1	1	0,06	0,01
C14:1-OH	1	2	2	10	6	4	5	6	11	4	3	3	8	6	6	4	0,14	0,02
C16:2	3	3	0	2	3	1	[illegible]	2	2	0	1	2	1	1	[illegible]	1	0,05	0,85
C16:1-OH/C14:1-DC	2	5	2	12	6	5	6	12	7	3	4	8	8	5	7	7	0,19	0,04
C16-OH	9	12	9	9	7	6	[illegible]	8	4	5	5	6	6	6	4	9	0,23	0,02
C18:2	10	12	15	9	6	5	7	10	3	5	5	8	[illegible]	7	4	[illegible]	0,90	0,02
C16	28	47	52	48	25	19	24	46	28	11	13	28	36	35	30	33	0,44	0,27
C18:3-OH/C16:3-DC	2	3	2	5	3	4	5	4	4	3	2	4	4	3	2	5	0,11	0,02
C18:2-OH/C16:2-DC	2	1	3	4	4	3	[illegible]	5	5	2	3	4	2	2	3	5	0,23	0,05
C20:2	6	12	11	8	5	2	3	5	3	2	2	4	[illegible]	3	3	5	0,15	0,02
C20	13	25	21	16	8	13	8	7	7	5	6	10	13	4	[illegible]	13	0,51	0,02
C22:5	2	4	1	1	0	1	0	1	0	1	0	2	[illegible]	2	2	[illegible]	0,01	0,00
C22:3	2	6	5	5	[illegible]	3	[illegible]	3	4	5	1	4	1	1	2	2	0,15	0,44
C22:2	5	7	3	3	3	3	4	3	3	1	2	1	4	2	3	2	0,05	0,04
C22	2	6	7	3	3	4	4	5	5	2	2	2	3	2	3	2	0,00	0,32

Green: Lower acycarnitine concentration; Red: Higher acylcarnitine concentration

Grey: Significant (t-test)
